# Supplementary material for: Flexibility and modulation of translation initiation in enterovirus genomes
Source: PLoS Pathog. 2026 Feb 9;22(2):e1013967. doi: 10.1371/journal.ppat.1013967 (PMC12904569; doi:10.1371/journal.ppat.1013967)
Supplement: S1 Fig — Columns show (1) the number of sequences with the given pattern of AUG triplets in the given BLASTCLUST cluster; (2) the positions of AUG triplets in the 20 nt 5′ of the SL-VI AUG; (3) the positions of AUG triplets in the 20 nt 3′ of the SL-VI AUG; and (4) the representative sequence of the relevant BLASTCLUST cluster (as in S3 Fig). Note that different sequences in the same BLASTCLUST cluster may harbour different non-SL-VI AUG configurations, and so the representative sequence does not necessarily contain the displayed AUG configuration. Sequences containing any ambiguous nucleotide codes (e.g., “N”, “R”, etc) in the 20 nt 5′ or 20 nt 3′ of the SL-VI AUG, or sequences with incomplete coverage of this region, were removed. Sequences without any non-SL-VI AUG triplets in this region are also not shown. Note that the smallest distance between the SL-VI AUG and the ppAUG is 21 nt (in some rhinoviruses); thus none of the displayed AUG triplets corresponds to the ppAUG. (DOCX) [file ppat.1013967.s001.docx]

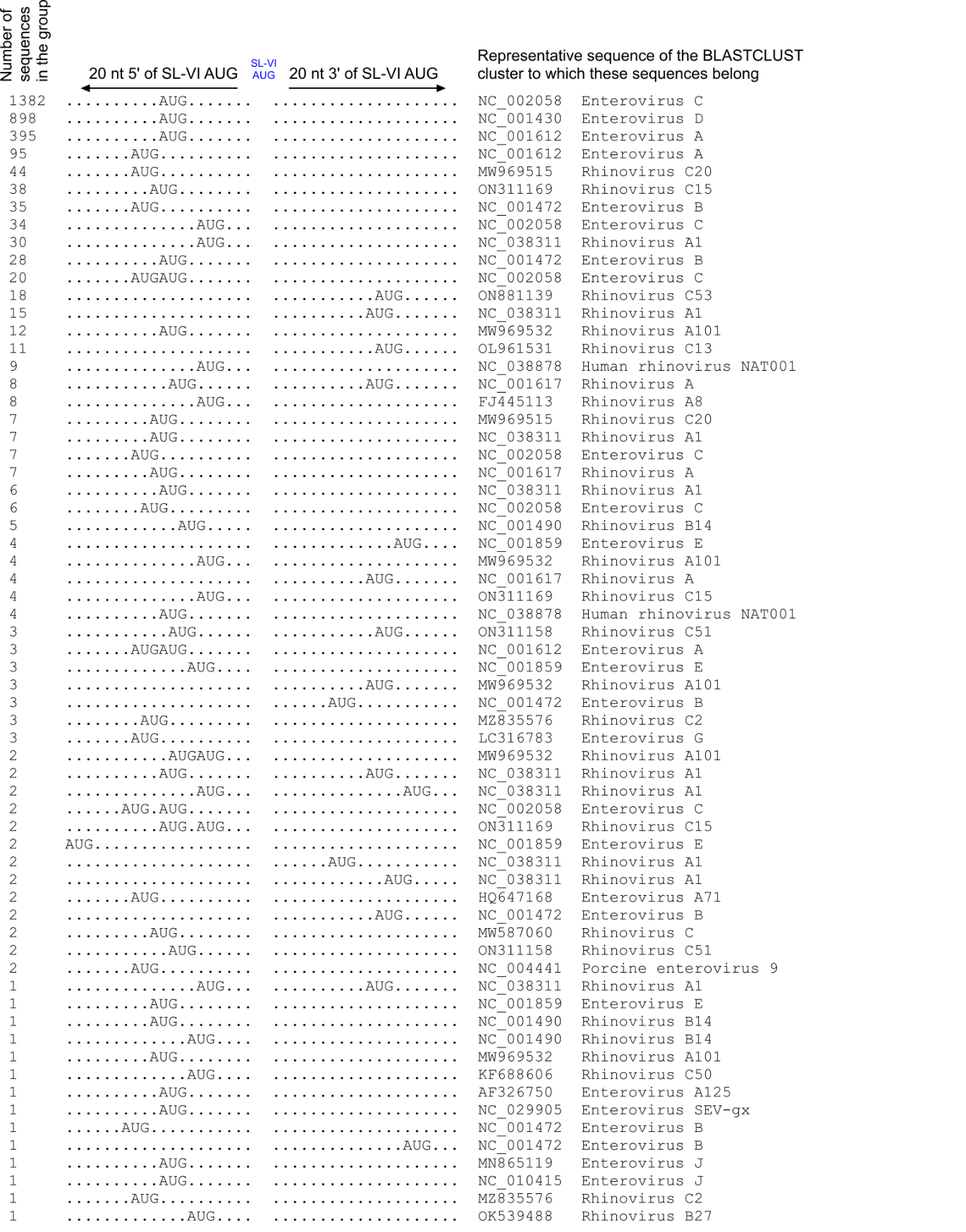


**S1 Fig. Analysis of AUG triplets in regions flanking the SL-VI AUG in 9347 enterovirus sequences.** Columns show (1) the number of sequences with the given pattern of AUG triplets in the given BLASTCLUST cluster; (2) the positions of AUG triplets in the 20 nt 5′ of the SL-VI AUG; (3) the positions of AUG triplets in the 20 nt 3′ of the SL-VI AUG; and (4) the representative sequence of the relevant BLASTCLUST cluster (as in Figure S3). Note that different sequences in the same BLASTCLUST cluster may harbour different non-SL-VI AUG configurations, and so the representative sequence does not necessarily contain the displayed AUG configuration. Sequences containing any ambiguous nucleotide codes (e.g. "N", "R", etc) in the 20 nt 5′ or 20 nt 3′ of the SL-VI AUG, or sequences with incomplete coverage of this region, were removed. Sequences without any non-SL-VI AUG triplets in this region are also not shown. Note that the smallest distance between the SL-VI AUG and the ppAUG is 21 nt (in some rhinoviruses); thus none of the displayed AUG triplets corresponds to the ppAUG.
