# Supplementary material for: Flexibility and modulation of translation initiation in enterovirus genomes
Source: PLoS Pathog. 2026 Feb 9;22(2):e1013967. doi: 10.1371/journal.ppat.1013967 (PMC12904569; doi:10.1371/journal.ppat.1013967)
Supplement: S2 Fig — Amino acid sequences of all ORFs that start with a non-SL-VI AUG within the 20 nt 5′ and 20 nt 3′ of the SL-VI AUG and that fulfill the following criteria: (a) encoded peptide ≥ 40 amino acids, (b) encoded peptide not contiguous with the polyprotein peptide, and (c) distance between the non-SL-VI AUG and the ppAUG ≥ 120 nt. The sequence WIGHP that is conserved in some enterovirus UP proteins is highlighted in blue. Transmembrane helix (TMH) predictions are highlighted in red (20–50% confidence), yellow (50–80% confidence) or green (>80% confidence); underlined sequences represent predicted N-terminal signal peptides (Phobius predictions). Columns show (1) BLASTCLUST cluster; (2) NCBI accession; (3) ‘Y’ if the non-SL-VI AUG ORF meets the more stringent Lulla et al. (2019) [1] uORF criteria (i.e., if the ORF beginning at the AUG and including the first in-frame stop codon overlaps the ppORF by at least 1 nt; is not in-frame with the ppORF; and contains at least 150 nt upstream of the ppAUG), otherwise ‘N’; and (4) the amino acid sequence of the ORF. For the purpose of cross-referencing with S3 Fig, the ‘representative sequences’ for the relevant clusters are: #1 NC_001612 Enterovirus A, #2 NC_002058 Enterovirus C, #3 NC_001472 Enterovirus B, #25 NC_010415 Enterovirus J, and #40 AF326750 Enterovirus A125. Note that some of these ORFs also have an in-frame SL-VI AUG (e.g., EF015017 – here, the SL-VI AUG corresponds to the methionine at position 5). (DOCX) [file ppat.1013967.s002.docx]

#1 MH933859 Y MAAYGDNLRLLSYSYWIGHPVTNRHIIYLFVGFVPLNSLSYQTLLYILKSNYLKQLWELKYLHRNQVHMRMQI

#1 MH118028 Y MAAYGDNLRLLSYSYWIGHPVNSRDIIYLFVGFTKFNITSYTTLLYIILLNNKKRKWELKCLLRRPGLTRIKMLLLMVLP

#1 MH118030 Y MAAYGDNLRLLSYSYWIGHPVNSRDIIYLFVGFTKLNITSYTTLLYIILLNNRKRKWELKCLLKRPDLTRTRMLLLTVLP

#1 MH118031 Y MAAYGDNLRLLSYSYWIGHPVTSRDIIYLFVGFVKLDTTTLKSLLFITQLNIRKRKWELKSQLRRLDLTRIKT

#1 AY697461 Y MAAYGDNLRLLSYSYWIGHPVTNRDLIYLFVGFTKLNITTFKTLLLIIQLNSEKRKWELKSQLRKLDRMRIKTSLQVDPL

#1 MG253032 Y MAAYGDNLRLLSYSYWIGHPVTNRDIIYLFVGFIRLNTITFKTLLYIIQLNSRKRKWELKSQPKRLDLTRTKT

#1 MG253033 Y MAAYGDNLRLLSYSYWIGHPVTNRDIIYLFVGFIRLNTITFKTLLYIIQLNSRKRKWELKSQPKRLDLMRTRT

#1 MG253035 Y MAAYGDNLRLLSYSYWIGHPVTNRDIIYLFVGFIRLNTITFKTLLYIIQLNSRKGKWELKSQPKRLDLTRTRT

#1 AY697459 Y MAAYGDNLRLLSYSYWIGHPVNSRDIIYLFVGFTTLNITNYRTLLYILLLNNRKRKWELKCPLKRPDRMRIRMLLPMALQ

#1 KT277550 Y MAAYGDNLRLLSYSYWIGHPVNSRDIIYLFVGFTTLNITNHRTLLYILLLNNRKRKWELKCPLKRPGHMRIRTLLPMVLQ

#1 AY697458 Y MAAYGDNLRLLSYSYWIGHPVNSRDIIYLFVGFIKLNITSYTTLLYIVLLNNRKRKWELKCLLRKPGLTRTKMLLLMVPP

#1 AY773285 Y MAAYGDNLRLLSYSYWIGHPVTNRDLAYLFVGFTKLDIVTFKTLLYIIQLNSRKRKWELKFQPKRLDLMRTRT

#1 JX390655 Y MAAYGDNLRLLSYSYWIGHPVNSRDIIYLFVGFTKLNITSYTTLLYIIQLNSEKRKWELKSQPKRLDLMRIRT

#1 KU355877 Y MAAYGDNLRLLSYSFWIGHSVTNRDLVYLFVGFTKLNTITLKTLLLIIQLNSRKRKWELRYQPRKLDLMRTRISLRVDQL

#1 JX390656 Y MAAYGDNLRLLSYSYWIGHPVNSRDIIYLFIGFTKLNITSYTTLLYIIQLNSRKRKWELKSQPKRLDLMRTRT

#1 AB192877 Y MAAYGDNLRLLSYSYWIGHPVTNRDIIYLFVGFVKLDTTAFNSLLFITQLNNRKRKWELKSQPRKLDLTRTRT

#1 AY697460 Y MAAYGDNLRLLSYSYWIGHPVTNRDIIYSFVGFVKLDSTTFKSLLFIIQLNNRKRKWELKSQPRKLDLTRTRT

#1 JX390654 Y MAAYGDNLRLLSYSYWIGHPVNSRDIIYLFVGFTKFNITSYTTLLYIIQLNSRKRKWELKSQPKRLDLMRTRT

#1 MG253034 Y MAAYGDNLRLLSYSYWIGHPVTNRDIIYLFVGFVRLNTITFKTLLYIIQLNSRKRKWELKSQPKRLDLTRTRT

#1 ON809571 Y MAAYGDNLRLLSYSYWIGHPVTNKHIIYLFVGFVPLNTLSYQTLLYILGLNYSKQQWELRCPHRSQDRMKMRIWPLADLL

#1 JF905564 Y MAAYGDNLRLLSYSYWIGHPVNSRDIIYLFVGFTKLNITSYTTLLYIILLNSKKRKWELKCLLKRPGLTRIRMLLLMVPP

#1 MH118029 Y MAAYGDNLRLLSYSYWIGHPVNSRDIIYLFVGFTKLNITSYTTLLYIILLNNRKRKWELKCLLKRPDLTKTRMLLLTVLP

#2 MN914206 Y MAAYGDNHRLLSYSDWIGHPVRKRDIIFLFVAFTPLNSFTPNLIKTVLLIRSVYHNGCASFITKGRGTRKHQCGYRWFNSKLHHY

#2 AB828290 N MAAYGDNLRLLSYSYWIGHPVIYRDLRTNPPFYNTHLLINLIIHNGGSGVHSKVWIARKPECCCWWFYHQLHHYKLLQG

#2 KX932039 N MAAYGDNLRLLSYSCWIGHPVIYRDLRTNPPFYNTHLLINLTIQNGSSSVHSEIWITREPESCCWRFHHQLYHHKLLQRQC

#2 KC785523 N MLMVTIVLISCCHYSRSVCNYKNKIISYYHPSRSVHNYKNEIFCHNGCSGICSEQWNTRKQKHSH

#2 MF990294 N MAAYGDNLRLLSYSIWIGHPVISKATHLSYCFTVFIYLLHPSNHNGSSSVHSEIWITREPECCSWWIHY

#2 MT432142 N MAAYGDNQRLLPQSELDWPSGECCVRYTTVCWNHCVSFTSHLTN

#2 OK570211 Y MAAYGDNLRLLSYSNWIGHPVRVRDIVYLFVAFAPLNTYTPSLIRIILLIRSAYHNGCTSFISKGRSP

#2 OK570194 Y MAAYGDNLGLLSYSNWIGHPVRVRDIIYLFVAFTPLNTYTPNLIKTVLLIRSLYHNGCTSFISEGGST

#2 OM963010 N MAAYGDNLRLLSYSCWIGHPVNCRDLNIYSFLCITYSLNISVQP

#2 LS451300 Y MAAYGDNLGLLSYSNWIGHPVRVRDIIYLFVAFTPLDTNTPILIRAVILIRSIHHNGCTSFISKGGST

#2 OK570210 Y MAAYGDNLRLLSYSNWIGHPVRIRDIIYLFVAYTPLNSYTPDLIKVVLLIRSIHHNGCASFISEGGST

#2 LS451301 Y MAAYGDNLRLLSYSDWIGHPVRIRDIIYLFVAFTPLNSYTPSLIKAVLLIRSAYHNGCTGFISKGGST

#2 ON383157 Y MAAYGDNLRLLSYSCWIGHPVKVKDIVYLFVAFTPLNNRTLSLIKIVLLLRAIYHNGCTGFIPKGGGT

#2 KC344834 N MAAYGDNQRLLSYSFWIGHPVIFETITFTCLTTQLSTLLHSHSHNGSSSVHTKVWFS

#2 KC785524 N MLMVTIVLISCCHCSRSVCNYENKTISCYHPPRSVHNHKNKIFCHNGCSGICSEQWNPRKQKHSHWWFNHQLHYY

#2 OP410421 N MAAYGDNQRLLSYSELDWPSGECCVRYTTVCWNHCVSFTSHLTN

#2 MG571859 N MAAYGDNLRLLSYSYWIGHPVIFESDICLAISLHLLTYNTANSQWELKCPLRNLDHMRIKTWLLVVPLLITPP

#2 MZ092702 N MLMVTIVLISCCHCSHSVCNHENKTISCYHPSRSVYNHKNEFFCHNGCSGICSEQWNPRKQKHSHWWLNHQLHYY

#2 MK250423 N MAAYGDNLRLLSYSYWTGHPVILKVILALLFHCICLFTILQTHNGSSSVHSEIRIT

#2 AF499635 N MAAYGDNLRLLSYSFWIGHPVIFEINIPSLLLHSTHLFITLL

#2 JX174176 N MAAYGDNLRLLSYSVWIGHPVTLKAIYLTHCLTEFTYLLHLYNNNGSSSVYSEVWIT

#2 JX174177 N MAAYGDNLRLLSYSVWIGHPVTSKAIYLTHCLTEFTYLLHLYNNNGSSSVYSEVWIT

#2 PP461545 Y MAAYGDNLRLLSYSYWIGHPVTNRDLIYLFVGFTKLNITNFKTLLFIIQLNNRKRKWELKYHHRKWVLMRTLM

#2 KJ170436 Y MWLLMVTITDCYHKANWIGHPVKVRFIIYLFAGFAPLSVFTLSTISTVISIRQLYHNGCSGFITESGRT

#2 KJ170510 Y MLMVTITDCYHKANWIGHPVKVRFIIYLFAGFAPLSVFTLSTISTVISIRQLYHNGCSGFITESGRT

#2 AF499641 N MAAYGDNLGLLSYSFWIGHPVTSNVQVVYSLNFLNTFSVVEL

#2 KR815824 N MLMVTIVLISCCHCSHSVCNYKNKTISCYHPSRSVYNHKNEFFCYNGCSGICSE

#2 JX982253 N MLMVTIVLISCCQYSRSVCNYKNKIISYYNPSRSVHNYENEIFCHNGCSGICSEQWNTRKQKHSHWWFNH

#2 JX982254 N MLMVTIVLISCCHYSRSVCNYKNKIISYYNPSRSVHNYENEIFCHNGCSGICSEQWNTRKQKHSHWWFNH

#2 JX982255 N MLMVTIVLISCCHYSRSVCNYKNKIISYYNPSRSVHNYENEIFCHNGCSGICSEQWNTRKQKHSHWWFNH

#2 JX982256 N MLMVTIVLISCCHYSRSVCNYKNKIISYYHPSRSVHNYKNEIFCHNGCPGICSEQWNTRKQKHSHWWFNH

#2 JX982257 N MLMVTIVLISCCHYSRSVCNYKNKIISYHHPSRSVHNYKNEIFCHNGCSGICSEQWNTRKQKHSHWWFNH

#2 JX982258 N MLMVTIVLISCCHYSRSVCNYKNKIISYYNPSRSVHNYENEIFCHNGCSGICSEQWNTRKQKHSHWWFNH

#2 JX982259 N MLMVTIVLISCCHYSRSVCNYKNKIISYYHPSRSVHNYKNEIFCHNGCSGICSEQWNTRKQKHSHWWFNH

#2 AB686524 N MLMVTIVLISCCHCSRSVCNYKNKTISCYHPPRSVHNHKNEIFCHNGRSSICSEQWNPRKQKHSHWWFNHQLHYY

#2 MZ092704 N MLMVTIVLISCCHCSHSVCNHENKTISCYHPSRSAYNHKNEFFCHNGCSGICSEQWNPRKQKHSHWWLNHQLHYY

#2 MH484166 Y MWLLMVTIIGCYHKANWIGHPVRVKHIIYLFVGFTPLSVFTPDVIRVVLLIRSFYHNGSSSIIPEGRST

#2 OP137321 N MWLLMVTIIDCYHKANWIGHPVKYKHIIYLLVGFISLTNLPLA

#2 JX275107 Y MAAYGNNHRLLSKSELDWPSSVNQINYSLVCWIRSRNVLLFNLLKLFEDRILVSQWELKYHPKK

#2 EF015017 Y MWLLMVTIIDCYHKANWIGHPVKYKHLIYLLVGFTPLTQFTPSIISTVLLVRHYHRYGCTGLISESGRS

#2 EF015015 Y MWLLMVTIIDCYHKASWIGHPVKYKHIIYLFVGFTPLTQFTPSIIIIVLLIRHQYQYGRTGLISESGCS

#2 EF015012 Y MAAYGDNLRLLSYSNWIGHPVRVRDIIYLFVAFTPLSTNTVTLIRIVLLIRLTHHYGCTSFISEGGCT

#2 EF555644 Y MAAYGDNLGLLSYSDWIGHPVRIKDIVYLFVAFTPLNKNTLSLIKVVLLIRSAYHNGCTSLISEGRGTRKHQRGHRWFNRKLHYHQLLQRFG

#2 EF015030 Y MAAYGAITDCYHKANWIGHPVKYKHIIYIFVGFTLLTHSSPKLIYTVLVIRNYHFSYGSSSIHSKDWGPREPECGCKRVHNQLYNHQLL

#3 KX981987 N MERLLPYSYWIGHPVTNRAIIYLFVRFIPLSLKEVKTLQFIVKLNTAKWELKYQRKRLGHMRPG

#25 AF414373 N MAAYGDNLTLLPYSCWVGHPDFVIKPFPRALTFNTFVNFFKSLLLVLKY

#40 AF326750 Y MAVYGDNLEYSDCHHKLLGLANRSIIYYFVGFVPLTPIIYKILLRFLLANLRYQRWVHKFLHNNLELMKTLMLQQEDQPYTTPPSTTTKTLMQHQQTSKIFHRTLQSLHNQWWMP

**S2 Fig. Non-SL-VI AUG ORFs with length at least 40 codons.** Amino acid sequences of all ORFs that start with a non-SL-VI AUG within the 20 nt 5′ and 20 nt 3′ of the SL-VI AUG and that fulfill the following criteria: (a) encoded peptide ≥ 40 amino acids, (b) encoded peptide not contiguous with the polyprotein peptide, and (c) distance between the non-SL-VI AUG and the ppAUG ≥ 120 nt. The sequence WIGHP that is conserved in some enterovirus UP proteins is highlighted in blue. Transmembrane helix (TMH) predictions are highlighted in red (20–50% confidence), yellow (50–80% confidence) or green (>80% confidence); underlined sequences represent predicted N-terminal signal peptides (Phobius predictions). Columns show (1) BLASTCLUST cluster; (2) NCBI accession; (3) 'Y' if the non-SL-VI AUG ORF meets the more stringent Lulla *et al.* (2019) ^1^ uORF criteria (i.e. if the ORF beginning at the AUG and including the first in-frame stop codon overlaps the ppORF by at least 1 nt; is not in-frame with the ppORF; and contains at least 150 nt upstream of the ppAUG), otherwise 'N'; and (4) the amino acid sequence of the ORF. For the purpose of cross-referencing with Figure S3, the 'representative sequences' for the relevant clusters are: #1 NC_001612 Enterovirus A, #2 NC_002058 Enterovirus C, #3 NC_001472 Enterovirus B, #25 NC_010415 Enterovirus J, and #40 AF326750 Enterovirus A125. Note that some of these ORFs also have an in-frame SL-VI AUG (e.g. EF015017 – here, the SL-VI AUG corresponds to the methionine at position 5).
