# Supplementary material for: Flexibility and modulation of translation initiation in enterovirus genomes
Source: PLoS Pathog. 2026 Feb 9;22(2):e1013967. doi: 10.1371/journal.ppat.1013967 (PMC12904569; doi:10.1371/journal.ppat.1013967)
Supplement: S3 Fig — The ppORF amino acid sequences of 9347 enterovirus sequences were clustered with BLASTCLUST using an 80% identity threshold, a representative sequence was selected from each of the 41 clusters, the ppORF amino acid sequences were aligned with MUSCLE, and a phylogenetic tree (left) was estimated with MrBayes (see Methods). Upstream AUG and upstream ORF statistics were calculated for each cluster. “distance ≥ 120 nt” refers to the distance between the upstream AUG and the ppAUG. “Non-SL-VI AUG uORF complements SL-VI uORF” indicates that the non-SL-VI AUG uORF and the SL-VI AUG uORF share the same termination codon [note, in the one case in the NC_001472 cluster, the non-SL-VI AUG is 3 codons 3′ of the SL-VI AUG and the resulting uORF fails the Lulla et al. (2019) [1] uORF criterion that the uORF initiation codon should be at least 150 nt upstream of the ppAUG]. “Non-SL-VI AUG uORF supplants SL-VI uORF” indicates that the non-SL-VI AUG uORF fulfills the Lulla et al. (2019) [1] uORF criteria, but the ORF beginning with the SL-VI AUG does not. (DOCX) [file ppat.1013967.s003.docx]

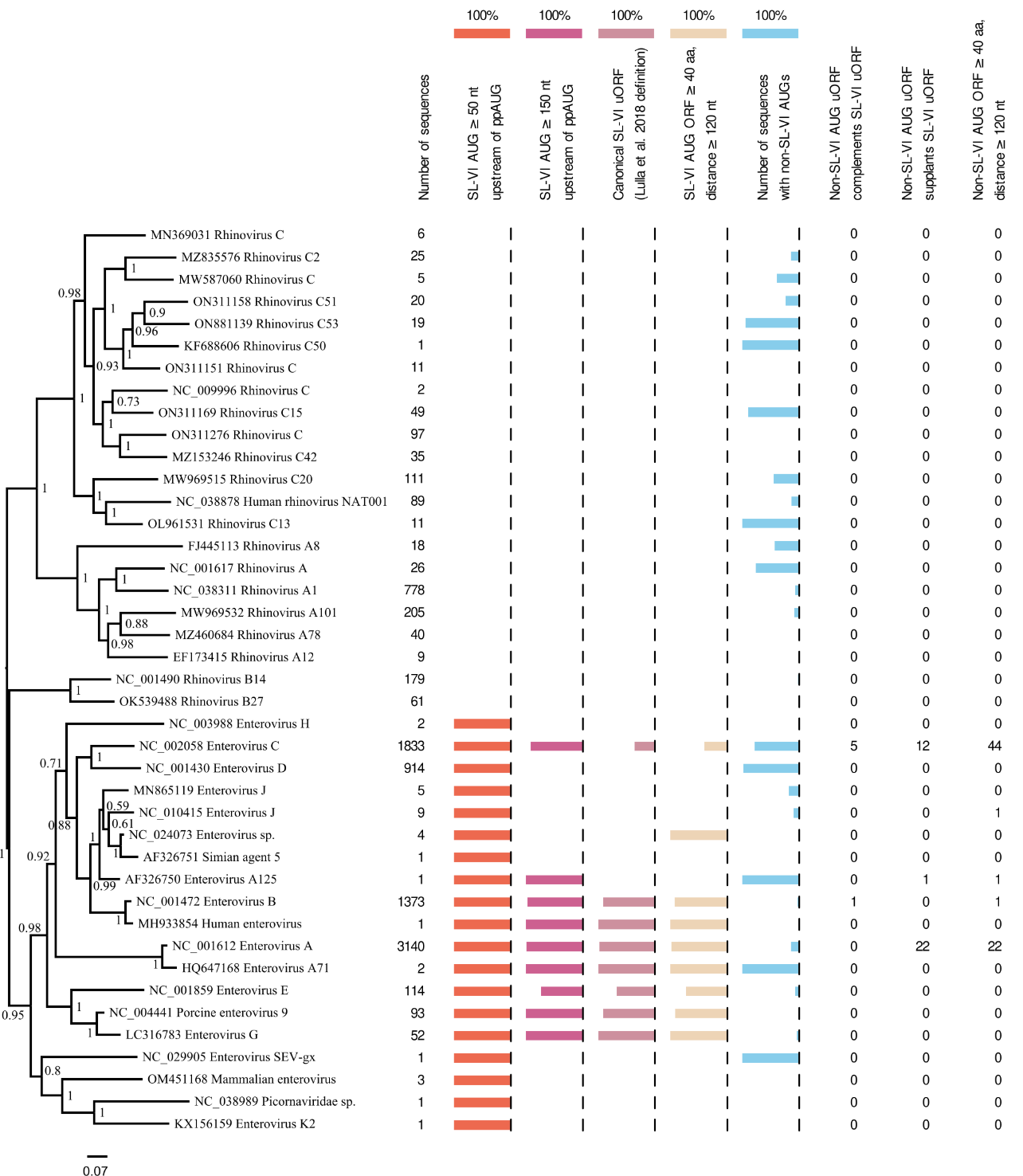


**S3 Fig. uORF statistics for different clusters of enterovirus sequences.** The ppORF amino acid sequences of 9347 enterovirus sequences were clustered with BLASTCLUST using an 80% identity threshold, a representative sequence was selected from each of the 41 clusters, the ppORF amino acid sequences were aligned with MUSCLE, and a phylogenetic tree (left) was estimated with MrBayes (see Methods). Upstream AUG and upstream ORF statistics were calculated for each cluster. "distance ≥ 120 nt" refers to the distance between the upstream AUG and the ppAUG. "Non-SL-VI AUG uORF complements SL-VI uORF" indicates that the non-SL-VI AUG uORF and the SL-VI AUG uORF share the same termination codon [note, in the one case in the NC_001472 cluster, the non-SL-VI AUG is 3 codons 3′ of the SL-VI AUG and the resulting uORF fails the [Lulla *et al.* (2019)](https://doi.org/10.1038/s41564-018-0297-1) uORF criterion that the uORF initiation codon should be at least 150 nt upstream of the ppAUG]. "Non-SL-VI AUG uORF supplants SL-VI uORF" indicates that the non-SL-VI AUG uORF fulfills the [Lulla *et al.* (2019)](https://doi.org/10.1038/s41564-018-0297-1) uORF criteria, but the ORF beginning with the SL-VI AUG does not.
