# Supplementary material for: Flexibility and modulation of translation initiation in enterovirus genomes
Source: PLoS Pathog. 2026 Feb 9;22(2):e1013967. doi: 10.1371/journal.ppat.1013967 (PMC12904569; doi:10.1371/journal.ppat.1013967)
Supplement: S4 Fig — (A) After first mapping RPFs to host RefSeq mRNAs, those mapping to annotated transcripts with exactly one upstream AUG (uAUG) and where the uAUG was within 200 nt upstream of the main AUG (mAUG) and not in the first 30 nt of the transcript, were selected. Next, RPFs whose 5′ end (+12 nt offset) mapped to the A of the uAUG or mAUG were quantified. Due to host-cell shut-off and the limited number of suitable host mRNAs used, the numbers of uAUG- and mAUG-mapping RPFs were often small, but useful numbers were obtained at least for the 5 h p.i. LTM libraries (marked in red). (B) As for panel A but for the CVA13 virus uAUG and mAUG codons. (C) Data from the 5 h p.i. LTM libraries in panel A, subdivided by uAUG initiation context: all contexts, strong contexts (G at −3 and +4, or A at −3), medium contexts (G at −3 or G at +4), and poor contexts (other contexts). As expected, there is a modest increase in uAUG:mAUG occupancy ratios with increasing strength of the uAUG initiation context. (D) Histograms of the 5′-end mapping positions of RPFs (no + 12 nt offset applied) relative to uAUG and mAUG codons, summed over the selected set of host mRNAs. RPFs whose 5′ ends map to the 1st, 2nd or 3rd positions of codons are shown in purple, blue or yellow, respectively. In all panels, only 27–29 nt reads were used. (DOCX) [file ppat.1013967.s004.docx]

**
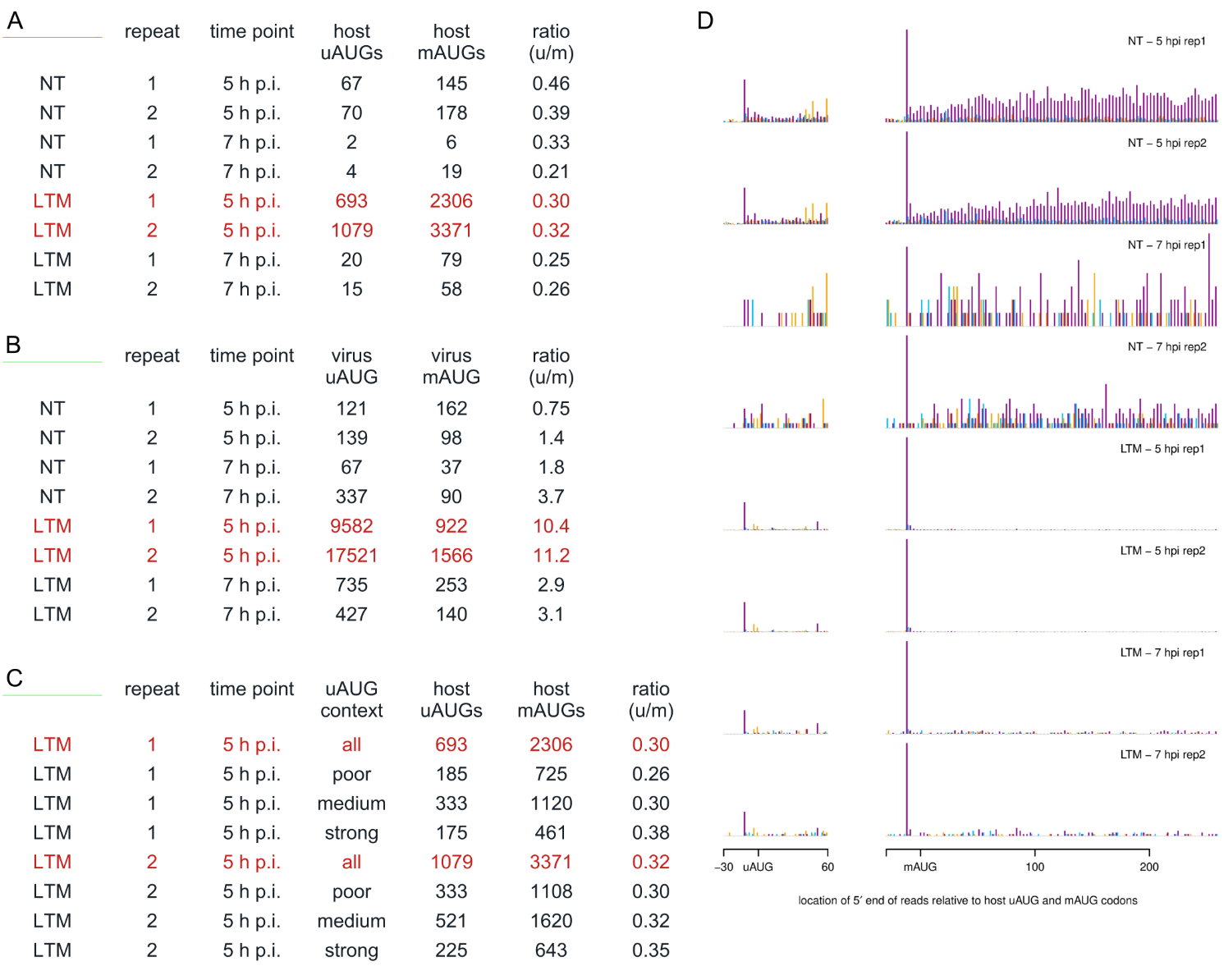
**

**S4 Fig. Relative RPF occupancy at upstream and main AUG codons.** (**A**) After first mapping RPFs to host RefSeq mRNAs, those mapping to annotated transcripts with exactly one upstream AUG (uAUG) and where the uAUG was within 200 nt upstream of the main AUG (mAUG) and not in the first 30 nt of the transcript, were selected. Next, RPFs whose 5′ end (+12 nt offset) mapped to the A of the uAUG or mAUG were quantified. Due to host-cell shut-off and the limited number of suitable host mRNAs used, the numbers of uAUG- and mAUG-mapping RPFs were often small, but useful numbers were obtained at least for the 5 h p.i. LTM libraries (marked in red). (**B**) As for panel A but for the CVA13 virus uAUG and mAUG codons. (**C**) Data from the 5 h p.i. LTM libraries in panel A, subdivided by uAUG initiation context: all contexts, strong contexts (G at −3 and +4, or A at −3), medium contexts (G at −3 or G at +4), and poor contexts (other contexts). As expected, there is a modest increase in uAUG:mAUG occupancy ratios with increasing strength of the uAUG initiation context. (**D**) Histograms of the 5′-end mapping positions of RPFs (no +12 nt offset applied) relative to uAUG and mAUG codons, summed over the selected set of host mRNAs. RPFs whose 5′ ends map to the 1st, 2nd or 3rd positions of codons are shown in purple, blue or yellow, respectively. In all panels, only 27-29 nt reads were used.
