# Supplementary material for: Flexibility and modulation of translation initiation in enterovirus genomes
Source: PLoS Pathog. 2026 Feb 9;22(2):e1013967. doi: 10.1371/journal.ppat.1013967 (PMC12904569; doi:10.1371/journal.ppat.1013967)
Supplement: S5 Fig — (A-B) Histograms of the 5′-end mapping positions of RPFs relative to annotated initiation and termination sites, summed over all host mRNAs. Reads whose 5′ ends map to the 1st, 2nd or 3rd positions of codons are shown in purple, blue or yellow, respectively. (C) Length distributions for Ribo-Seq reads mapping to virus (red) or host mRNA (purple) coding regions; upper panels – LTM, lower panels – NT. (D) Phasing of 5′ ends of Ribo-Seq reads that map to the virus or host mRNA coding regions. (E-F) Ribosome profiles for the CVA13 genome at 5 and 7 hpi for cells treated with lactimidomycin (E) or untreated (F). Histograms of the 5′-end mapping positions of RPFs, with a + 12 nt offset to map the approximate P-site position, in reads per million mapped reads (RPM), smoothed with a 15-nucleotide running mean filter. For panels A, B, D, E and F, only 27–29 nt reads were used. (DOCX) [file ppat.1013967.s005.docx]

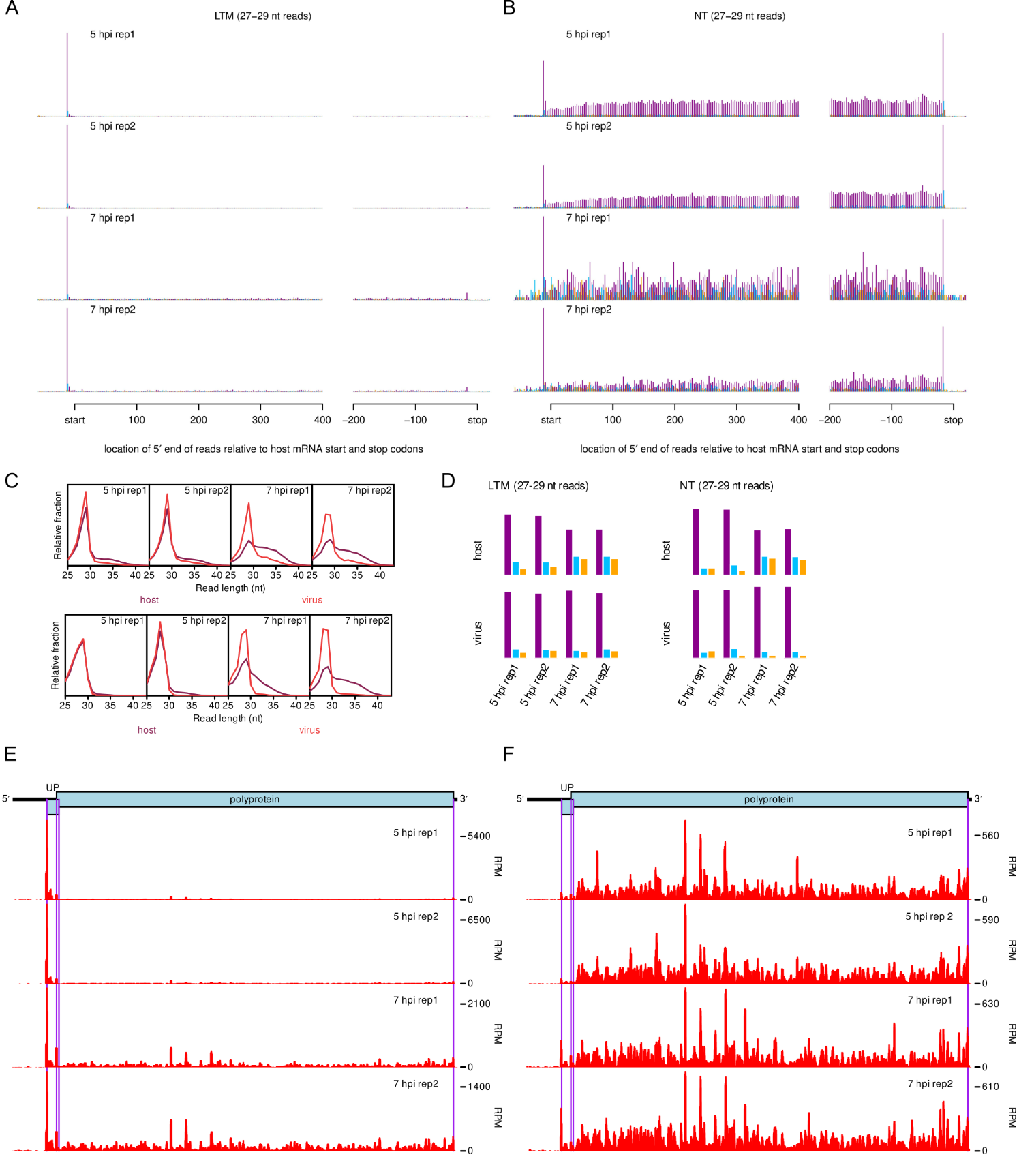


**S5 Fig. Assessment of ribosome profiling quality for lactimidomycin-treated (LTM) and non-treated (NT) CVA13 libraries.** (**A-B**) Histograms of the 5′-end mapping positions of RPFs relative to annotated initiation and termination sites, summed over all host mRNAs. Reads whose 5′ ends map to the 1st, 2nd or 3rd positions of codons are shown in purple, blue or yellow, respectively. (**C**) Length distributions for Ribo-Seq reads mapping to virus (red) or host mRNA (purple) coding regions; upper panels – LTM, lower panels – NT. (**D**) Phasing of 5′ ends of Ribo-Seq reads that map to the virus or host mRNA coding regions. (**E-F**) Ribosome profiles for the CVA13 genome at 5 and 7 hpi for cells treated with lactimidomycin (E) or untreated (F). Histograms of the 5′-end mapping positions of RPFs, with a +12 nt offset to map the approximate P-site position, in reads per million mapped reads (RPM), smoothed with a 15-nucleotide running mean filter. For panels A, B, D, E and F, only 27–29 nt reads were used.
