# Supplementary material for: Flexibility and modulation of translation initiation in enterovirus genomes
Source: PLoS Pathog. 2026 Feb 9;22(2):e1013967. doi: 10.1371/journal.ppat.1013967 (PMC12904569; doi:10.1371/journal.ppat.1013967)
Supplement: S6 Fig — (A) Nucleotide (left) and uuORF-encoded protein (right) sequences in enterovirus CVA1, where the uORF is truncated and the uuORF can potentially rescue UP expression. Transmembrane helix (TMH) predictions are highlighted in yellow (50–80% confidence) or green (>80% confidence). Depending on the frame used, ORFs are highlighted in blue (uuORF), and purple or orange (uORF). (B) Schematic representation of the CVA1 IRES dVI region with the uuORF (blue), uORF and ppORF (orange) start and stop codons annotated. (C) Schematic representation of the CVA13, CVA1, and EV-A90 reporters used to measure translation in three frames (Fig 5B–5D). The 5′ UTR from all three viruses was inserted into dual luciferase-expressing constructs (Fig 5A) so that WT sequence is preserved (including start and stop codons) until ppORF start codon. Then the sequence of ppORF is replaced by firefly luciferase in all three frames. (DOCX) [file ppat.1013967.s006.docx]

**
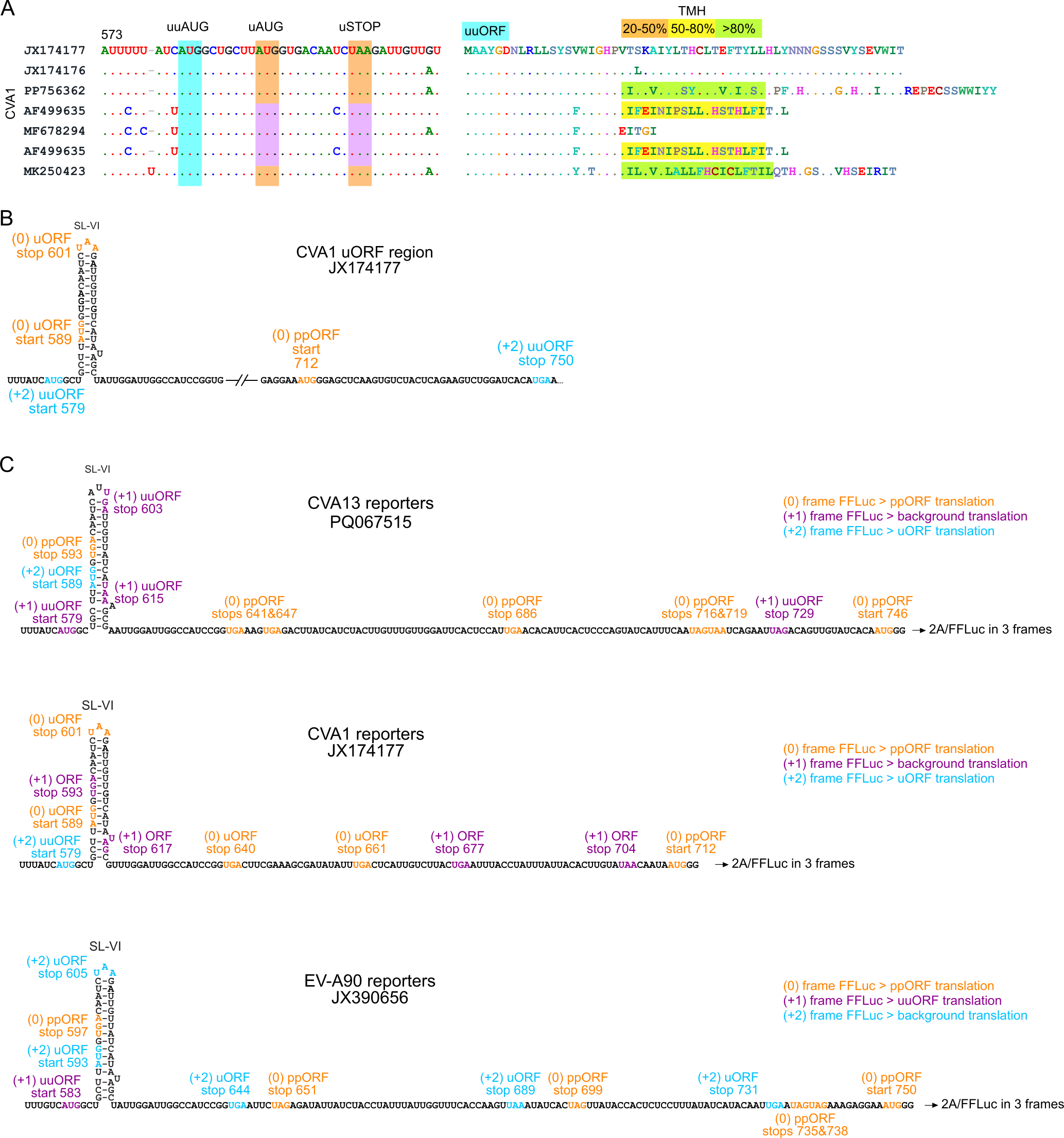
**

**S6 Fig. Enterovirus CVA1 sequences that encode a UP-like protein from an alternative upstream AUG codon.** (**A**) Nucleotide (left) and uuORF-encoded protein (right) sequences in enterovirus CVA1, where the uORF is truncated and the uuORF can potentially rescue UP expression. Transmembrane helix (TMH) predictions are highlighted in yellow (50–80% confidence) or green (>80% confidence). Depending on the frame used, ORFs are highlighted in blue (uuORF), and purple or orange (uORF). (**B**) Schematic representation of the CVA1 IRES dVI region with the uuORF (blue), uORF and ppORF (orange) start and stop codons annotated. (**C**) Schematic representation of the CVA13, CVA1, and EV-A90 reporters used to measure translation in three frames (Fig. 5B-D). The 5′ UTR from all three viruses was inserted into dual luciferase-expressing constructs (Fig. 5A) so that WT sequence is preserved (including start and stop codons) until ppORF start codon. Then the sequence of ppORF is replaced by firefly luciferase in all three frames.
