# Supplementary material for: Flexibility and modulation of translation initiation in enterovirus genomes
Source: PLoS Pathog. 2026 Feb 9;22(2):e1013967. doi: 10.1371/journal.ppat.1013967 (PMC12904569; doi:10.1371/journal.ppat.1013967)
Supplement: S7 Fig — (A) The growth of CVA13 viruses in organoid line derived from patient 2. (B) Representative sequencing chromatograms of RT-PCR products from viruses derived from the final time points in Fig 7A and 7C. (C) Differentiation of human intestinal organoid cultures. Results of qRT-PCR showing fold change in transcript levels in differentiated duodenum organoids (days 1–4) relative to transcript levels in undifferentiated organoids (day 0). The GAPDH transcript was used for normalization. LGR5, a stem cell marker, leucine-rich repeat-containing G-protein coupled receptor 5; ALP, a mature enterocyte marker, alkaline phosphatase; Villin, an epithelial cell marker. Plotted data represent means ± s.d.; n = 3. (DOCX) [file ppat.1013967.s007.docx]

**
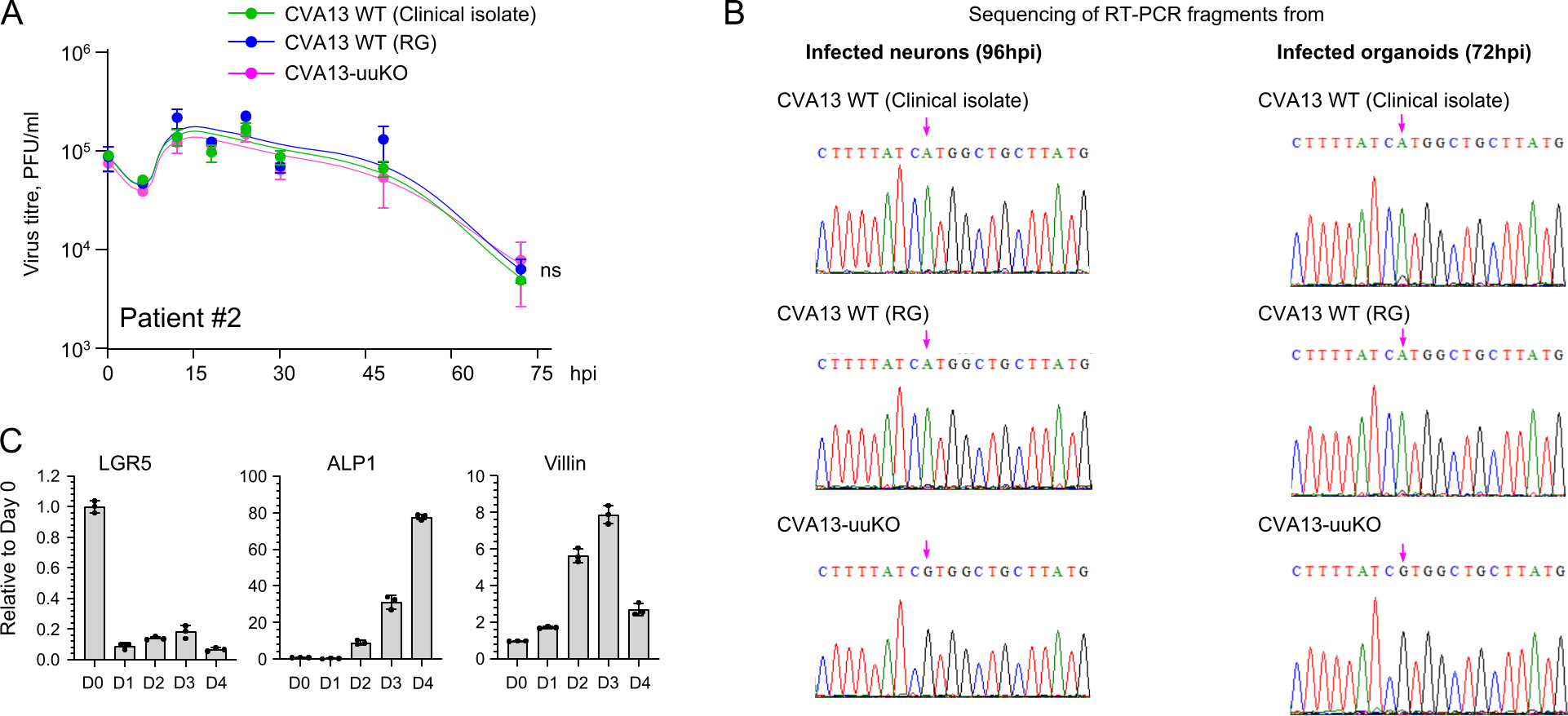
**

**S7 Fig. Validation of organoid and neuron infection experiments.** (**A**) The growth of CVA13 viruses in organoid line derived from patient 2. (**B**) Representative sequencing chromatograms of RT-PCR products from viruses derived from the final time points in Fig. 7A and 7C. (**C**) Differentiation of human intestinal organoid cultures. Results of qRT-PCR showing fold change in transcript levels in differentiated duodenum organoids (days 1-4) relative to transcript levels in undifferentiated organoids (day 0). The GAPDH transcript was used for normalization. LGR5, a stem cell marker, leucine-rich repeat-containing G-protein coupled receptor 5; ALP, a mature enterocyte marker, alkaline phosphatase; Villin, an epithelial cell marker. Plotted data represent means ± s.d.; n = 3.
