# Supplementary material for: Flexibility and modulation of translation initiation in enterovirus genomes
Source: PLoS Pathog. 2026 Feb 9;22(2):e1013967. doi: 10.1371/journal.ppat.1013967 (PMC12904569; doi:10.1371/journal.ppat.1013967)
Supplement: S8 Fig — Analysis of IRES activities for CVA13 5′-3′ UTR reporters in the three frames in HeLa and intestinal HIEC6 cells, with and without virus RNA, at 8 h.p.t. Statistical analysis was conducted using two-tailed t-tests; * p value ≤ 0.05; ns, non significant. (DOCX) [file ppat.1013967.s008.docx]

**
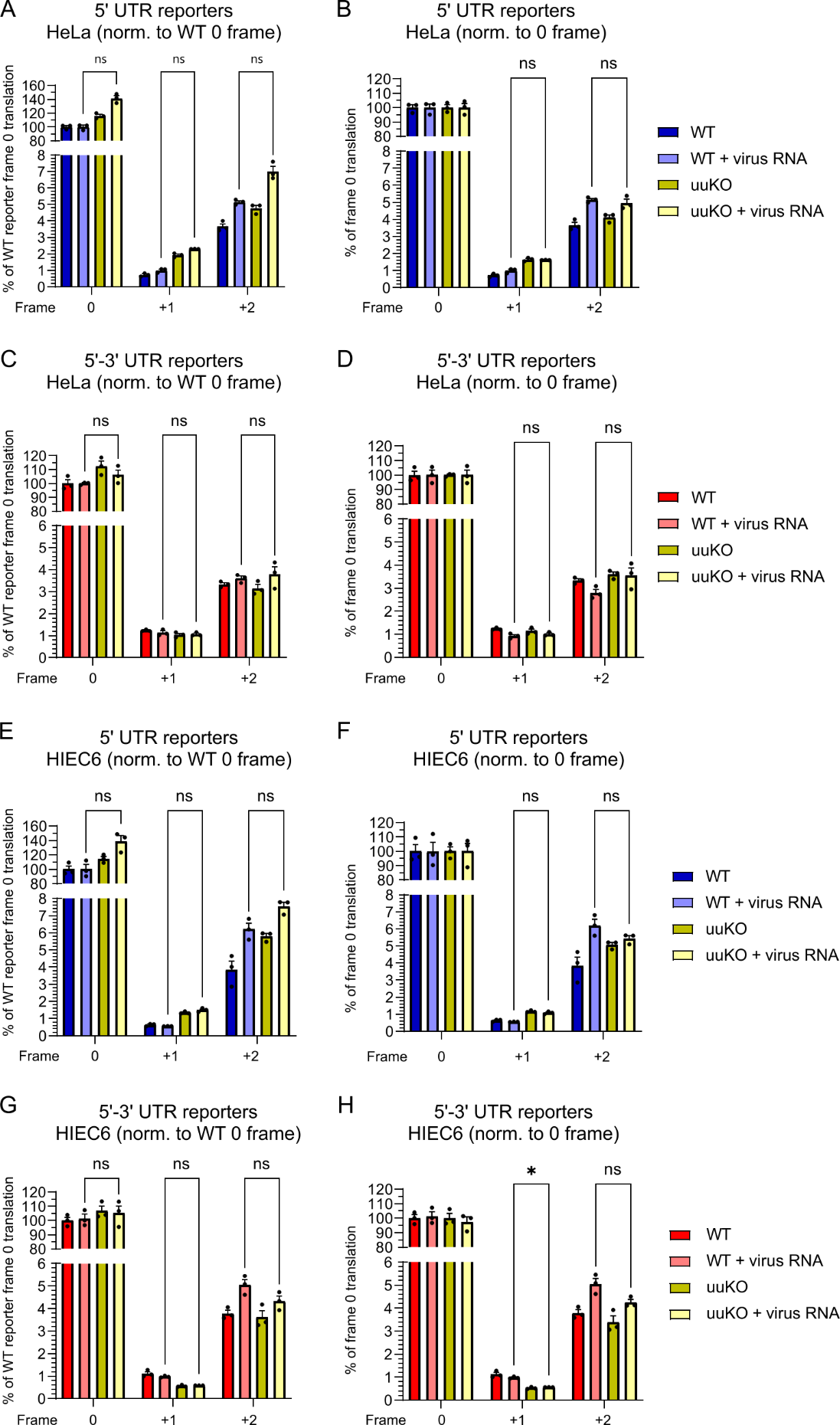
**

**S8 Fig. Full set of expression assays performed for 5′UTR and 5′-3′UTR reporters.** Analysis of IRES activities for CVA13 5′-3′ UTR reporters in the three frames in HeLa and intestinal HIEC6 cells, with and without virus RNA, at 8 h.p.t. Statistical analysis was conducted using two-tailed *t*-tests; * *p* value ≤ 0.05; ns, non significant.
