# Supplementary material for: Flexibility and modulation of translation initiation in enterovirus genomes
Source: PLoS Pathog. 2026 Feb 9;22(2):e1013967. doi: 10.1371/journal.ppat.1013967 (PMC12904569; doi:10.1371/journal.ppat.1013967)
Supplement: S9 Fig — A region from 20 nt 5′ of the SL-VI AUG to 30 nt 3′ of the SL-VI AUG was extracted. All AUG codons are shown in the three reading frames. Stop codons (STP) are only shown if they terminate an ORF initiated by one (or more) of the displayed AUGs. Other nucleotides are shown as “n” if they are part of one of these ORFs, otherwise as “.”. Sequences with incomplete coverage of this region, or any ambiguous nucleotide codes (“R”, “N”, etc) in this region were removed, leaving 9333 sequences. Number of sequences (#seqs) list the number of sequences with the given configuration of displayed AUGs, stops, and short ORFs. (DOCX) [file ppat.1013967.s009.docx]

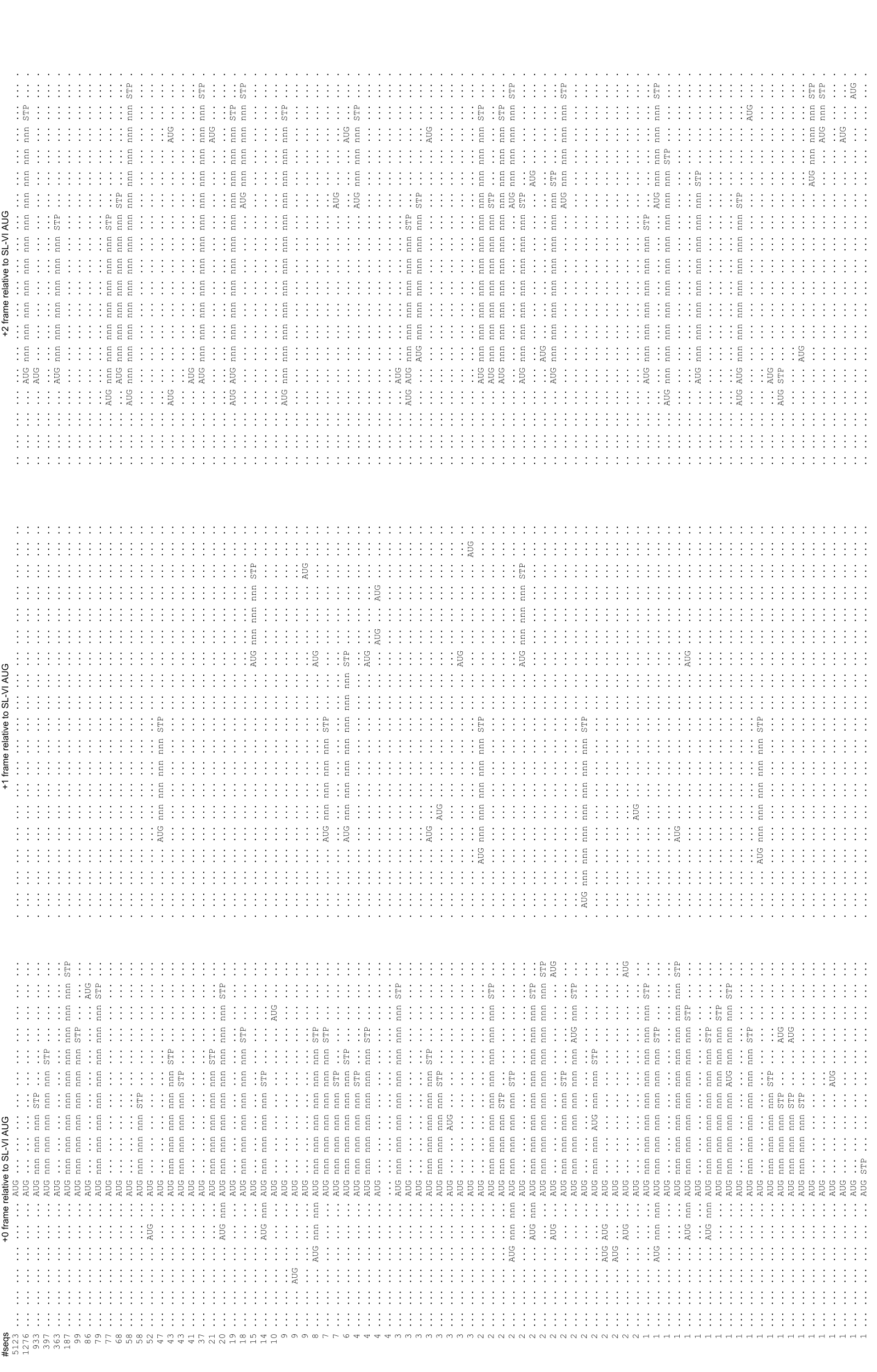


**S9 Fig. Mapping positions of short ORFs in SL-VI region.** A region from 20 nt 5′ of the SL-VI AUG to 30 nt 3′ of the SL-VI AUG was extracted. All AUG codons are shown in the three reading frames. Stop codons (STP) are only shown if they terminate an ORF initiated by one (or more) of the displayed AUGs. Other nucleotides are shown as "n" if they are part of one of these ORFs, otherwise as ".". Sequences with incomplete coverage of this region, or any ambiguous nucleotide codes ("R", "N", etc) in this region were removed, leaving 9333 sequences. Number of sequences (#seqs) list the number of sequences with the given configuration of displayed AUGs, stops, and short ORFs.
