# Supplementary material for: Flexibility and modulation of translation initiation in enterovirus genomes
Source: PLoS Pathog. 2026 Feb 9;22(2):e1013967. doi: 10.1371/journal.ppat.1013967 (PMC12904569; doi:10.1371/journal.ppat.1013967)
Supplement: S10 Fig — (A-B) Schematic representation of the CVA1 (A) and EV-A90 (B) reporters and their AUU mutants used to measure translation in three frames (Fig 8E–8F). (C) Cell viability during sodium arsenite (NaAs) treatment (8 hours post-transfection, 8 hours post-treatment). The data are normalized to the untreated transfected control (0 μM), and presented as mean ± SEM (n = 3). (DOCX) [file ppat.1013967.s010.docx]

**
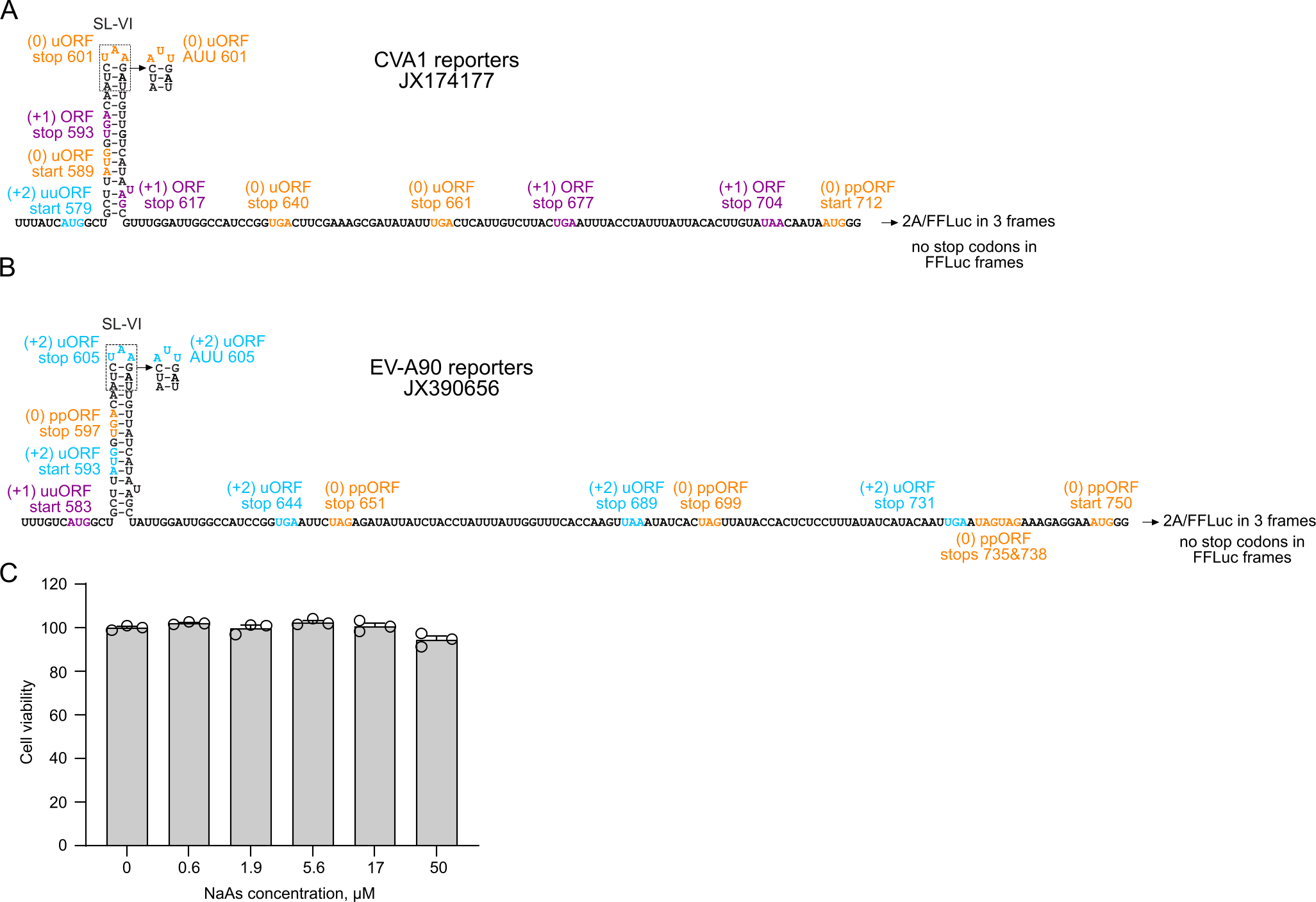
**

**S10 Fig. Supplementary data for Figure 8.** (**A-B**) Schematic representation of the CVA1 (A) and EV-A90 (B) reporters and their AUU mutants used to measure translation in three frames (Fig. 8E-F). (**C**) Cell viability during sodium arsenite (NaAs) treatment (8 hours post-transfection, 8 hours post-treatment). The data are normalized to the untreated transfected control (0 μM), and presented as mean ± SEM (*n* = 3).
