# Supplementary material for: Flexibility and modulation of translation initiation in enterovirus genomes
Source: PLoS Pathog. 2026 Feb 9;22(2):e1013967. doi: 10.1371/journal.ppat.1013967 (PMC12904569; doi:10.1371/journal.ppat.1013967)
Supplement: S1 Table — (DOCX) [file ppat.1013967.s011.docx]

**S1 Table**. Metadata associated with *E. alphacoxsackie* species.

| **Enterovirus A isolate sequence** | **Type** | **Isolation source** | **Isolation country** | **Year** | **Associated disease** | **Reference**  **PMID** |
| --- | --- | --- | --- | --- | --- | --- |
| KT277550 | A89 | stool | China | 2011 | contact of an AFP patient | 26685900 |
| AY697459 | A89 | stool | Bangladesh | 2000 | isolated from an AFP patient | 15659764 |
| MH118030 | A76 | N/A | India | 2011 | N/A | N/A |
| MH118029 | A76 | N/A | India | 2010 | N/A | N/A |
| MH118028 | A76 | N/A | India | 2010 | N/A | N/A |
| AY697458 | A76 | stool | Bangladesh | 1999 | isolated from an AFP patient | 15659764 |
| JF905564 | A76 | stool | China | 2004 | isolated from an AFP patient | 21755310 |
| MH118031 | A90 | N/A | India | 2010 | N/A | N/A |
| MH933859 | A90 | stool | Cameroon | 2014 | N/A | N/A |
| AB192877 | A90 | N/A | Cambodia | 2005 | isolated from an AFP patient | N/A |
| AY773285 | A90 | N/A | Netherlands | 2004 | isolated from an AFP patient | N/A |
| AY697460 | A90 | stool | Bangladesh | 1999 | isolated from an AFP patient | 15659764 |
| JX390654 | A90 | stool | China | 2001 | isolated from an AFP patient | 23081679 |
| JX390655 | A90 | stool | China | 2001 | isolated from an AFP patient | 23081679 |
| JX390656 | A90 | stool | China | 2003 | isolated from an AFP patient | 23081679 |
| MG253035 | A90 | stool | China | 2011 | isolated from an AFP patient | 29980696 |
| MG253034 | A90 | stool | China | 2011 | isolated from an AFP patient | 29980696 |
| MG253033 | A90 | stool | China | 2011 | isolated from an AFP patient | 29980696 |
| MG253032 | A90 | stool | China | 2011 | isolated from an AFP patient | 29980696 |
| KU355877 | A121 | stool | India | 2013 | healthy child | 27902407 |
| AY697461 | A91 | stool | Bangladesh | 2000 | isolated from an AFP patient | 15659764 |
| ON809571 | A119 | raw wastewater | France | 2015 | N/A | 37212710 |
| AF326750 | A125 | baboon stool |  | 1962 | N/A | 11773400 |

AFP – acute flaccid paralysis, N/A – no data available.
