# Supplementary material for: Flexibility and modulation of translation initiation in enterovirus genomes
Source: PLoS Pathog. 2026 Feb 9;22(2):e1013967. doi: 10.1371/journal.ppat.1013967 (PMC12904569; doi:10.1371/journal.ppat.1013967)
Supplement: S2 Table — (DOCX) [file ppat.1013967.s012.docx]

**S2 Table.** Metadata associated with *E. coxsackiepol* species.

| **Enterovirus C isolate sequence** | **Type** | **Isolation source** | **Isolation country** | **Year** | **Associated disease** | **Reference**  **PMID** |
| --- | --- | --- | --- | --- | --- | --- |
| EF015012 | EV-C99 | clinical isolate, CDC | Oklahoma, USA | 1985 | N/A | 19264596 |
| EF015030 | CVA21 | stool | Bangladesh | 2000 | AFP | 19264596 |
| EF555644 | EV-C99 | clinical isolate, CDC | Georgia, USA | 1984 | N/A | 19264596 |
| JX275107 | PV2 | PV2 outbreak | Nigeria | 2008 | paralytic disease (poliomyelitis) | 23408630 |
| LS451300 | EV-C99 | stool | Madagascar | 2003 | polio surveillance | 30323802 |
| LS451301 | EV-C99 | stool | Madagascar | 2003 | polio surveillance | 30323802 |
| MN914206 | EV-C99 | stool | Malawi | 2003 | surveillance | 32629843 |
| OK570194 | EV-C99 | stool | Madagascar | 2011 | polio surveillance | 36348312 |
| OK570210 | EV-C99 | stool | Madagascar | 2011 | polio surveillance | 36348312 |
| OK570211 | CVA20 | stool | Madagascar | 2011 | polio surveillance | 36348312 |
| ON383157 | EV-C99 | stool | Guatemala | 2020 | environmental water from human excretion | 35950869 |
| PP461545 | CVA24 | stool | Nepal | 2023 | N/A | N/A |

AFP – acute flaccid paralysis, N/A – no data available.
