# Supplementary material for: Flexibility and modulation of translation initiation in enterovirus genomes
Source: PLoS Pathog. 2026 Feb 9;22(2):e1013967. doi: 10.1371/journal.ppat.1013967 (PMC12904569; doi:10.1371/journal.ppat.1013967)
Supplement: S3 Table — (DOCX) [file ppat.1013967.s013.docx]

**S3 Table.** Host and virus read counts for Ribo-Seq samples.

| treatment | repeat | time point | total reads | host mRNA(+) | vRNA(+) | host mRNA(+) | vRNA(+) |
| --- | --- | --- | --- | --- | --- | --- | --- |
|  |  |  |  | all reads ≥25 nt | | 27–29 nt reads | |
| NT | 1 | 5 hpi | 7,816,493 | 337,247 | 202,634 | 182,022 | 142,629 |
| NT | 2 | 5 hpi | 6,651,515 | 393,300 | 296,507 | 220,144 | 226,696 |
| NT | 1 | 7 hpi | 11,437,523 | 32,125 | 23,185 | 13,893 | 16,891 |
| NT | 2 | 7 hpi | 9,203,363 | 102,111 | 85,105 | 40,605 | 63,126 |
| LTM | 1 | 5 hpi | 8,427,129 | 190,159 | 72,899 | 114,910 | 53,860 |
| LTM | 2 | 5 hpi | 11,635,282 | 278,943 | 107,787 | 162,110 | 75,024 |
| LTM | 1 | 7 hpi | 9,384,830 | 46,725 | 22,484 | 16,822 | 13,186 |
| LTM | 2 | 7 hpi | 7,860,105 | 35,459 | 20,473 | 13,951 | 12,975 |
